# Lifestyle characteristics in adolescent female football players: data from the Karolinska football Injury Cohort

**DOI:** 10.1186/s13102-022-00603-1

**Published:** 2022-12-14

**Authors:** Anne Fältström, Eva Skillgate, Nathan Weiss, Henrik Källberg, Victor Lyberg, Markus Waldén, Martin Hägglund, Martin Asker, Ulrika Tranaeus

**Affiliations:** 1 Department of Health Promotion Science, Musculoskeletal & Sports Injury Epidemiology Center, Sophiahemmet University, Stockholm, Sweden; 2 Unit of Physiotherapy, Department of Health, Medicine and Caring Sciences, Linköping University, Linköping, Sweden; 3 Region Jönköping County, Rehabilitation Centre, Ryhov County Hospital, Jönköping, Sweden; 4 Unit of Intervention and Implementation for Worker Health, Institute of Environmental Medicine, Karolinska Institutet, Stockholm, Sweden; 5 Unit of analysis, Department of Public Health, Analysis and Data Management, Public Health Agency of Sweden, Stockholm, Sweden; 6 Unit of Public Health, Department of Health, Medicine and Caring Sciences, Linköping, Sweden; 7 GHP Ortho Center Skåne, Malmö, Sweden; 8 Sport Without Injury ProgrammE (SWIPE), Linköping University, Linköping, Sweden; 9 Naprapathögskolan, Scandinavian College of Naprapathic Manual Medicine, Stockholm, Sweden; 10 Department of Physiology, Nutrition, Biomechanic, Sport Performance & Exercise Research & Innovation Center - Stockholm, SPERIC-S, The Swedish School of Sport and Health Sciences, Stockholm, Sweden

**Keywords:** Coping, Passion, Soccer, Stress, Youth

## Abstract

**Background:**

Normative values of lifestyle characteristics in adolescent female football players may be used by clinicians and coaches to take actions because the potential important for well-being, performance on the pitch, and risk of injury. The aim was to report descriptive characteristics of lifestyle factors in adolescent female football players and potential changes over 1 year.

**Methods:**

We included 419 adolescent competitive female football players from 12 clubs and 27 teams (age 14 ± 1 years, range 12–17 years) and 286 were followed over 1 year. The players completed an extensive questionnaire regarding demographics, football-related factors, and lifestyle factors including tobacco consumption, alcohol use, medicine intake, eating and sleeping habits, well-being, stress, coping, and passion. Baseline data are presented for the total cohort and separately for 4 age groups (12, 13, 14, and 15–17 years).

**Results:**

12% skipped breakfast, 8% skipped lunch and 11% used protein supplements several days per week. 16% slept less than 8 h/night, 8% had impaired sleep with daytime consequences, and 22% stated that they were tired in daily activities several days per week. 32% experienced stress some or most days/week and 24% were classified as having psychological distress. Medicine intake (23% vs. 34%), skipping breakfast or lunch several days per week (10% vs. 47% and 20 vs. 33%), tiredness (20% vs. 27%), stress (26% vs. 40%), and psychological distress (27% vs. 37%) increased significantly (*P* = 0.031 to < 0.001) at the 1-year follow-up.

**Conclusion:**

Many adolescent female football players skip breakfast and lunch, have insufficient sleep, experience stress and are classified as having psychological distress. These factors increased over 1 year.

## Background

Football (soccer) is the most popular female sport in the world with more than 13 million registered female players and more than 3 million female players under the age of 18 years [1]. For young girls, participation in football has a positive influence on both physical and psychological well-being, social and peer support, and perception of the school environment [2], but it also comes with a high risk of injury [3]. Various medical problems, life stress, daily hassles, poor dietary intake, and poor sleeping habits have been shown to negatively affect well-being, performance on the pitch and the risk of injury [4, 5]. There has been increased focus recently on a healthy lifestyle with a good diet, proper sleeping habits, no or little use of tobacco and alcohol, the use of psychological techniques (e.g. self-efficacy, imagery, and motivation), how athletes behave in different stressful situations (i.e. coping strategies) and the association with overall health, performance and risk of injury [5, 6].

Surveys are performed for monitoring health and risk factors in public health, and data collected are used in planning, evaluation, and resource allocation by decision-makers and health care planners [7]. To our knowledge, there is no such monitoring among adolescent athletes. It is important for clinicians and all sports entities i.e., coaches and their staff, to know the normal characteristics of a defined population in terms of, for example, sport, age, and sex [8, 9], and be aware of what to expect and potentially take actions of potential importance for the athlete’s performance on the pitch, well-being, and the risk of injury.

The aim of this study was to report normative data and possible changes over 1 year for football-related factors and lifestyle factors, including tobacco consumption, alcohol use, eating and sleeping habits, well-being, stress, coping, and passion among adolescent female football players.

## Methods

### Design

This study is based on the Karolinska football Injury Cohort study [10].

### Participants

The regional football district of Stockholm, Sweden, consists of 140 teams with approximately 2520 female players aged 13–19 years. Adolescent female football players from 28 teams in the 2 highest divisions for girls were invited to participate; 27 teams agreed to participate and were included in the study. Players, parents, legal guardians and coaches in each team were invited to a meeting and were given oral and written information about the study [10]. All players signed written consent and the legal guardians also signed if players were younger than 16 years. The study was approved by the Swedish Ethical Review Authority (Dnr 2016/1251-31/4).

### Procedures

An extensive questionnaire (in Swedish) regarding demographics, football-related factors and lifestyle factors, including tobacco consumption, alcohol use, medicine intake, eating and sleeping habits, well-being, stress, coping, and passion for sport was distributed to the players at baseline [10]. The players were instructed to fill out the questionnaire at home [10]. The questionnaires were checked by a research assistant to minimize the risk of misunderstood questions or missing answers. The players were included consecutively during 4 years (2016–2019). A similar questionnaire was sent out at the 1-year follow-up.

#### Demographics and football-related factors

First, there were some general questions about age, height, body mass, and menarche. There were also specific questions about football participation, such as years playing organized football, dominant limb (defined as preferred kicking limb), usual playing position, performing any injury prevention training regularly (e.g., Knee Control, HarmoKnee, or FIFA 11+) [11] and training and match exposure (average weekly estimate for the preceding 6 months).

#### Tobacco consumption, alcohol use, eating habits and medicine intake

Questions about tobacco use, alcohol (“How often do you drink at least one glass of alcohol [liquor, wine, beer, strong cider, mixed drink]”?), and eating habits (“How often do you skip breakfast/lunch/dinner?” and “How often do you use supplements such as protein shakes/bars?”), and each question was rated on a five-point scale (never, rarely, few times/year, a few times/month, several times/week, and every day) according to the Stockholm Public Health Cohort survey were included [7]. Questions about medication intake (“Do you use any medications, e.g., for asthma, pain killers, and birth control pills?”) were also asked.

#### Sleeping habits

The definition of impaired sleep used in this study is based on international diagnostic criteria: difficulty initiating and/or maintaining sleep accompanied by daytime consequences [12]. The players reported how they experienced their sleep using 3 questions [13]. Two of the questions, “Do you have difficulty falling asleep?” and “Do you wake up several times at night and sometimes have difficulties going back to sleep?”, were used to evaluate impaired sleep or good sleep. A third question “Do you feel very tired during your daily activities?” was also used to evaluate any daytime consequences. The questions were rated on the same 5-point scale as described earlier, ranging from 1 (never) to 5 (always, every day). If they answered 4 (several times per week) or 5 (every day) on either of the first 2 questions and also rated 4 or 5 on the third question, they were defined as having impaired sleep accompanied by a daytime consequence. Impaired sleep without a daytime consequence was defined as having problems in initiating or maintaining sleep several times per week or every day, but having no daytime consequence [13].

#### Well-being, stress, sport psychology, coping and passion

##### General health questionnaire-12, stress and sport psychology

The General Health Questionnaire (GHQ) is a validated questionnaire measuring well-being and current mental health [14]. GHQ-12 contains 12 items covering areas such as anxiety, depressed mood, social function and loss of confidence referring to the past several weeks. Each question was rated on a 4-point scale with the options “more/better than usual” to “much less/much worse than usual” or “not at all” to “much more than usual” depending on the question. GHQ-12 gives a total score of 36 (0-1-2-3 on each question) with higher scores indicating worse mental health [15]. GHQ-12 is also reported with a total score of 12 (0-0-1-1 on each question), where a score ≥ 3 denotes psychological distress [7]. A single question about stress was also used (“How often do you feel stressed?”) with the options ranging from “never” to “most days of the week” [7]. The players were asked if they had any education in sport psychology (“Have you received education in sports psychology and/or mental training?”), and any regular contact with a sport psychology consultant (“Do you regularly meet any sport psychology consultant/mental training?”).

##### Brief COPE

Coping strategies indicate how people behave in different stressful situations and are often measured with self-reported questionnaires such as the Brief COPE [16]. The Brief COPE consists of 14 scales each with 2 items, measuring conceptually differentiable coping strategies. Response options are scored on a 4-point scale, measuring “How often” they had used the different coping strategies to cope with stress (“I have not been doing this at all” to “I have been doing this a lot”). Adaptive behaviours were captured in the items for active coping, emotional support, instrumental support, positive reframing, planning, humour, acceptance, and religion. The items on maladaptive behaviours consisted of self-distraction, denial, substance, behavioural disengagement, venting, and self-blame.

##### Passion scale

The Passion Scale consists of 14 questions [17], with a scale of 7 alternatives (1, do not agree at all; 7, very strongly agree) when referring to their football activity. One dimension consists of 7 harmonious passion items (e.g., “I am completely taken with this activity”) and the other dimension consists of 7 obsessive passion items (e.g., “I have almost an obsessive feeling for my activity”).

### Statistical analyses

All statistical analyses were performed in SPSS Statistics for Windows (IBM SPSS Statistics for Windows, Version 27.0. IBM, Armonk, NY). Descriptive statistics are presented as means ± standard deviation or numbers and proportions. The cohort was divided into age groups (12 years, 13 years, 14 years, and 15–17 years) and were followed over 1 year. Paired-sample t test was used for continuous data and McNemar’s test for nominal data to compare baseline with follow-up data. The significance level was set at *P* < 0.05. Effect sizes are presented as Cohen’s *d* for continuous data, where *d* = 0.2 indicates a small effect, *d* = 0.5 indicates a medium effect, and *d* = 0.8 indicates a large effect.

## Results

Forty players quit football during the follow-up period and 20 players changed teams. Characteristics and normative data for the 419 players overall and for 286 of 379 current players (response rate 75%) who answered also at the 1-year follow-up are presented in Table 1. The sub-cohort of players (n = 286) who responded to both baseline and follow-up questionnaires included players aged 12 years (n = 68, 24%), 13 years (n = 110, 38%), 14 years (n = 54, 19%), and 15–17 years (n = 54, 19%) at baseline. First, baseline normative data for the total cohort (n = 419) and then changes in data for the sub-cohort (n = 286) are presented.

**Table 1:** Characteristics for adolescent female football players at baseline and at the 1-year follow-up

	Total cohort (12–17 years)		Total cohort divided into age groups				Sub-cohort (12–17 years)^a^		Baseline versus Follow-Up	Effect Size
	n	n = 419	12 years (n = 97)	13 years (n = 158)	14 years (n = 91)	15–17 years (n = 73)	Baseline (n = 286)	Follow-Up (n = 286)	*P* Value	Cohen’s *d*
Age, years	418	13.9 ± 1.1	12.7 ± 0.2	13.4 ± 0.3	14.5 ± 0.3	15.8 ± 0.6	13.9 ± 1.2	14.9 ± 1.6		
Height, cm	417	163 ± 6.8	160 ± 6.4	161 ± 6.5	165 ± 5.7	168 ± 5.8	162.5 ± 6.8	165.5 ± 5.6	**< 0.001**	1.07
Body mass, kg	417	53 ± 9.0	48 ± 7.5	51 ± 7.7	57 ± 7.6	61 ± 7.6	53.2 ± 9.0	57.8 ± 7.7	**< 0.001**	1.08
Body mass index, kg/m^2^	416	20.1 ± 2.5	18.8 ± 2.2	19.6 ± 2.2	20.9 ± 2.4	21.8 ± 2.1	20.0 ± 2.5	21.1 ± 2.4	**< 0.001**	0.65
Menarche, n (%)	418	280 (67)	35 (36)	92 (58)	82 (90)	71 (97)	184 (64)	–	–	
Age at menarche	416	12.4 ± 1.0	11.9 ± 0.9	12.3 ± 0.9	12.7 ± 1.1	12.6 ± 1.1	12.5 ± 1.1	–	–	
Amenorrhea >3 months^b^, n (%)	412	46 (16)	4 (4)	17 (11)	9 (10)	16 (22)				
Years playing organized football	417	7.0 ± 2.2	6.0 ± 1.9	6.3 ± 1.8	7.5 ± 1.8	9.2 ± 1.7	7.0 ± 2.2	–	–	
Dominant leg, n (%)	419									
Right		399 (95)	95 (98)	149 (94)	87 (96)	68 (93)	270 (94)	–	–	
Left		17 (4)	2 (2)	8 (5)	4 (4)	3 (4)	13 (5)	–	–	
Both		3 (1)	0 (0)	1 (1)	0 (0)	2 (3)	3 (1)	–	–	
Playing position, n (%)	414								0.466	
Goalkeeper		34 (8)	6 (6)	11 (7)	9 (10)	8 (11)	19 (7)	21 (7)		
Defender		130 (31)	37 (38)	45 (29)	25 (28)	23 (32)	85 (30)	95 (33)		
Midfielder		168 (41)	29 (30)	79 (51)	36 (40)	24 (33)	117 (42)	109 (38)		
Forward		82 (20)	25 (26)	21 (13)	19 (21)	17 (24)	61 (22)	59 (21)		
Football matches/week, n	419	1.5 ± 0.6	1.6 ± 0.6	1.5 ± 0.6	1.4 ± 0.7	1.2 ± 0.6	1.5 ± 0.6	1.4 ± 0.7	0.387	–0.05
Football training h/week, n	419	5.0 ± 1.8	4.6 ± 1.5	5.0 ± 1.6	5.0 ± 1.7	5.4 ± 2.6	5.1 ± 1.9	5.1 ± 2.0	0.783	0.02
Other training with football team h/week	416	1.6 ± 1.4	1.4 ± 1.2	1.5 ± 1.3	1.5 ± 1.1	2.2 ± 1.8	1.7 ± 1.5	1.6 ± 1.2	0.365	–0.05
Injury prevention training, yes	419	320 (76)	77 (79)	121 (77)	65 (71)	57 (78)	223 (78)	199 (70)	**0.011**	
Prevention times/week	400	2.5 ± 1.3	2.5 ± 1.2	2.7 ± 1.2	2.2 ± 1.2	2.4 ± 1.4	2.6 ± 1.3	2.9 ± 1.4	**0.010**	0.21
1–2 times/week		157 (39)	36 (37)	48 (30)	39 (43)	34 (47)	107 (37)	70 (25)		
3–4 times/week		127 (32)	32 (33)	59 (37)	20 (22)	17 (23)	93 (33)	96 (34)		
>4 times/week		16 (4)	3 (3)	7 (4)	2 (2)	4 (5)	11 (4)	21 (7)		
Training with older players in the previous 6 months	416								**< 0.001**	
Every week		70 (17)	11 (12)	16 (10)	16 (18)	27 (37)	52 (18)	24 (8)		
Few times/every month		28 (7)	2 (2)	12 (8)	4 (4)	10 (14)	18 (6)	37 (13)		
Few times previous 6 months		99 (24)	26 (27)	38 (24)	23 (25)	12 (16)	56 (20)	90 (32)		
Never		219 (53)	56 (59)	91 (58)	48 (53)	24 (33)	157 (56)	133 (47)		
Match play with older players in the previous 6 months	418								0.509	
Every week		33 (8)	7 (7)	10 (6)	8 (9)	8 (11)	22 (8)	19 (7)		
Few times/every month		52 (12)	7 (7)	17 (11)	15 (16)	13 (18)	42 (15)	42 (15)		
Few times in the previous 6 months		124 (30)	29 (30)	46 (29)	25 (27)	24 (33)	79 (28)	92 (32)		
Never		209 (50)	54 (56)	84 (54)	43 (47)	28 (38)	142 (50)	133 (46)		
Other training (not football), n (%)	418	135 (32)	33 (34)	55 (36)	23 (25)	23 (32)	90 (32)	85 (30)	0.615	
Other training (not football), h/week	133	2.7 ± 2.0	2.5 ± 2.1	2.4 ± 1.7	2.7 ± 2.2	3.5 ± 2.4	2.8 ± 2.1	2.3 ± 1.5	**0.002**	−0.45

Values are reported as means ± standard deviations or n (%). *P* values in bold type are significant. Effect size measured as Cohen’s *d*, where *d* = 0.2 indicates a small effect, *d* = 0.5 indicates a medium effect, and *d* = 0.8 indicates a large effect. The values regarding training/match are weekly averages of the preceding 6 months. –, data not collected

^a^Missing value from 0–3 players for the different questions both at baseline and at follow-up

^b^The question: If you menstruation has become regular, has your period stopped for more than 3 months in a row, or longer?

In the total cohort (n = 419), 76% of the players stated that they performed an injury prevention training programme regularly. About half of the players had trained and played matches with older players in the previous 6 months. The proportion of players who used injury prevention training decreased (78% vs. 70%), but the frequency of use increased slightly (2.6 vs. 2.9 times/week) between baseline and 1-year follow-up.

Tobacco and alcohol use was rare. 12% skipped breakfast, 8% skipped lunch and 11% used protein supplements several times per week or every day (Table 2). A higher proportion skipped breakfast (10% vs. 17%) and lunch (7% vs. 12%) several times per week or every day and never used protein supplements (16% vs. 25%) at the 1-year follow-up compared with baseline. The proportion of players who used any medicine also increased from 23 to 34% at the 1-year follow-up compared with baseline.

**Table 2:** Lifestyle factors among adolescent female football players at baseline and at the 1-year follow-up

	Total cohort (12–17 years)		Total cohort divided into age groups				Sub-cohort (12–17 years)^a^		Baseline versus Follow-Up
	n	n = 419	12 years (n = 97)	13 years (n = 158)	14 years (n = 91)	15–17 years (n = 73)	Baseline (n = 286)	Follow-Up (n = 286)	*P* Value
Smoking	419								0.198
No, never		414 (99)	97 (100)	157 (100)	88 (97)	72 (99)	283 (99)	276 (97)	
Rarely, a few times/year		2 (0.5)	0 (0)	0 (0)	1 (1)	1 (1)	2 (1)	4 (1)	
A few times/month		2 (0.5)	0 (0)	0 (0)	2 (2)	0 (0)	1 (0)	3 (1)	
Several times/week		0 (0)	0 (0)	0 (0)	0 (0)	0 (0)	0 (0)	2 (0)	
Snus	418								0.513
Never		417 (99.5)	96 (99)	157 (100)	91 (100)	73 (100)	285 (100)	283 (99)	
Rarely, a few times/year		0 (0)	0 (0)	0 (0)	0 (0)	0 (0)	0 (0)	2 (1)	
Several times/week		1 (0.5)	1 (1)	0 (0)	0 (0)	0 (0)	1 (0)	1 (0)	
Drink at least one glass of alcohol^b^	415								**0.032**
Never		398 (96)	96 (100)	155 (98)	86 (96)	61 (85)	272 (96)	254 (90)	
Rarely, few times/year		7 (2)	0 (0)	1 (1)	1 (1)	5 (7)	6 (2)	10 (3.5)	
A few times/month		10 (2)	0 (0)	1 (1)	3 (3)	6 (8)	5 (2)	16 (6)	
Several times/week		0 (0)	0 (0)	0 (0)	0 (0)	0 (0)	0 (0)	1 (0.4)	
Every day		0 (0)	0 (0)	0 (0)	0 (0)	0 (0)	0 (0)	1 (0.4)	
Skipping breakfast	419								**0.012**
Never		197 (47)	47 (49)	72 (46)	46 (50)	32 (44)	145 (51)	118 (41)	
Rarely, few times/year		88 (21)	22 (23)	38 (24)	17 (18)	11 (15)	63 (22)	61 (21)	
A few times/month		81 (19)	13 (13)	32 (20)	16 (18)	20 (27)	49 (17)	60 (21)	
Several times/week		47 (11)	11 (11)	14 (9)	12 (13)	10 (14)	25 (9)	39 (14)	
Every day		6 (1)	4 (4)	2 (1)	0 (0)	0 (0)	4 (1)	8 (3)	
Skipping lunch	418								**0.001**
Never		193 (46)	49 (50)	76 (48)	39 (43)	29 (40)	137 (48)	105 (37)	
Rarely, a few times/year		106 (25)	19 (20)	38 (24)	29 (32)	20 (27)	78 (27)	77 (27)	
A few times/month		83 (20)	17 (18)	34 (22)	13 (14)	19 (26)	50 (18)	68 (24)	
Several times/week		35 (8)	11 (11)	10 (6)	9 (10)	5 (7)	20 (7)	30 (11)	
Every day		1 (0.2)	1 (1)	0 (0)	0 (0)	0 (0)	0 (0)	3 (1)	
Skipping dinner	414								**0.031**
Never		284 (69)	74 (77)	106 (68)	62 (68)	42 (59)	201 (71)	170 (60)	
Rarely, a few times/year		95 (23)	18 (19)	37 (24)	22 (24)	18 (25)	59 (21)	81 (29)	
A few times/month		33 (8)	4 (4)	13 (8)	7 (8)	9 (13)	19 (7)	25 (9)	
Several times/week		2 (0.5)	0 (0)	0 (0)	0 (0)	2 (3)	2 (1)	3 (1)	
Every day		0 (0)	0 (0)	0 (0)	0 (0)	0 (0)	0 (0)	2 (1)	
Using protein supplements	415								**0.030**
Never		73 (18)	24 (25)	23 (15)	12 (13)	14 (19)	44 (16)	70 (25)	
Rarely, a few times/year		109 (26)	28 (29)	35 (23)	24 (26)	22 (31)	75 (27)	66 (23)	
A few times/months		187 (45)	38 (39)	82 (53)	46 (51)	21 (29)	128 (45)	115 (40)	
Several times/week		42 (10)	7 (7)	13 (8)	8 (9)	14 (19)	32 (11)	32 (11)	
Every day		4 (1)	0 (0)	2 (1)	1 (1)	1 (1)	4 (1)	2 (1)	
Intake of any medication, yes	412	97 (23)	19 (20)	28 (18)	23 (25)	27 (37)	64 (23)	97 (34)	**< 0.001**
Asthma/allergy medicine		51 (12)	15 (15)	17 (11)	9 (10)	10 (14)	35 (12)	32 (11)	
Painkillers		19 (5)	1 (1)	7 (4)	5 (6)	6 (8)	13 (5)	20 (7)	
Birth control pills		10 (2)	0 (0)	1 (1)	4 (4)	5 (7)	4 (1)	18 (6)	
Vitamins		4 (1)	2 (2)	1 (1)	1 (1)	0 (0)	1 (0)	4 (1)	
Acne medicine		4 (1)	1 (1)	0 (0)	1 (1)	2 (3)	4 (1)	1 (0)	
Others		5 (1)	0 (0)	1 (1)	2 (2)	2 (3)	2 (3)	5 (2)	
Several medications		4 (1)	0 (0)	1 (1)	1 (1)	2 (3)	4 (1)	17 (6)	

Values are reported as n (%). *P* values in bold type are significant

^a^Missing value from 0–5 players for the different questions both at baseline and at follow-up

^b^The question: “How often do you drink at least one glass of alcohol (liquor, wine, beer, strong cider, mixed drink)?”

At baseline, 16% slept less than 8 h/night, 8% had impaired sleep with daytime consequences, and 22% stated that they were tired during the day every day or several times per week (Table 3). A higher proportion of players reported that they felt tired during the day every day or several times/week at the 1-year follow-up compared with baseline (20% vs. 27%).

**Table 3:** Sleeping habits among adolescent female football players at baseline and at the 1-year follow-up

	Total cohort (12–17 years)		Total cohort divided into age groups				Sub-cohort (12–17 years)^a^		Baseline vs. Follow-Up
	n	n = 419	12 years (n = 97)	13 years (n = 157)	14 years (n = 91)	15–17 (n = 73)	Baseline (n = 286)	Follow-Up (n = 286)	*P* value
Sleeping hours	402	8.3 ± 0.8	8.7 ± 0.8	8.4 ± 0.7	8.0 ± 0.9	7.8 ± 0.8	8.3 ± 0.8	–	–
6–7.5 h		65 (16)	5 (5)	15 (10)	23 (26)	22 (34)	44 (16)	–	
8 h		169 (42)	34 (35)	72 (47)	35 (40)	28 (43)	112 (40)	–	
8.5–10 h		168 (42)	58 (60)	65 (43)	30 (34)	15 (23)	123 (44)	–	
Sleeping problems: go back to sleep	419								0.111
Never		88 (21)	26 (27)	32 (20)	18 (20)	12 (16)	60 (21)	46 (16)	
Rarely, few times/year		138 (33)	31 (32)	51 (32)	34 (37)	22 (30)	92 (32)	86 (30)	
Some few times/months		129 (31)	23 (24)	51 (32)	27 (30)	28 (38)	95 (33)	108 (38)	
Several times/week		58 (14)	14 (14)	23 (15)	11 (12)	10 (14)	36 (13)	38 (13)	
Every day		6 (1)	3 (3)	1 (1)	1 (1)	1 (1)	3 (1)	7 (3)	
Sleeping problems: woke up	417								0.576
Never		160 (38)	34 (35)	64 (41)	33 (36)	29 (40)	108 (38)	109 (38)	
Rarely, few times/year		177 (42)	39 (41)	60 (38)	42 (46)	36 (49)	123 (43)	115 (40)	
Some few times/months		68 (16)	20 (21)	29 (18)	13 (14)	6 (8)	48 (17)	46 (16)	
Several times/week		10 (2)	3 (3)	4 (3)	2 (2)	1 (1)	5 (2)	12 (4)	
Every day		2 (1)	0 (0)	0 (0)	1 (1)	1 (1)	1 (0)	3 (1)	
Feel tired in daily activities	417								**0.025**
Never		47 (11)	16 (16)	22 (14)	8 (9)	1 (1)	31 (11)	21 (7)	
Rarely, few times/year		149 (36)	42 (43)	59 (38)	31 (34)	17 (23)	100 (35)	73 (25)	
Some few times/months		131 (31)	22 (23)	52 (33)	35 (39)	22 (30)	97 (34)	116 (41)	
Several times/week		70 (17)	16 (16)	20 (13)	10 (11)	24 (33)	44 (15)	59 (21)	
Every day		20 (5)	1 (1)	4 (2)	6 (7)	9 (12)	13 (5)	17 (6)	
Impaired sleep with daytime consequences	419	34 (8)	7 (7)	8 (5)	8 (9)	11 (15)	19 (7)	25 (9)	0.327
Impaired sleep without daytime consequence	419	31 (7)	8 (8)	18 (11)	4 (4)	1 (1)	21 (7)	26 (9)	0.522

Values are reported as mean, standard deviations or n (%). *P* values in bold type are significant. –, data not collected

^a^Missing value from 0–1 player for the different questions both at baseline and at follow-up. Seven missing answers at baseline regarding sleeping hours

According to GHQ-12, 24% were classified as having psychological distress and 32% stated that they were stressed some or most days/week (Table 4). A higher proportion of players were classified as having psychological distress (27% vs. 37%) and stated that they experienced stress some or most days/week (26% vs. 40%) at the 1-year follow-up compared with baseline.

**Table 4:** Well-being and passion among adolescent female football players at baseline and at the 1-year follow-up

	Total cohort (12–17 years)		Total cohort divided into age groups				Sub-cohort (12–17 years)^a^		Baseline versus Follow-Up	Effect Size
	n	n = 419	12 years (n = 97)	13 years (n = 157)	14 years (n = 91)	15–17 years (n = 73)	Baseline (n = 286)	Follow-Up (n = 286)	*P* Value	Cohen’s *d*
*General Health Questionnaire-12*										
0–36	398	10.9 ± 4.0	9.9 ± 3.6	10.9 ± 3.9	10.9 ± 4.4	12.4 ± 3.6	10.8 ± 4.1	12.1 ± 4.2	**< 0.001**	0.30
0–2 (no psychological distress)	401	303 (76)	78 (84)	117 (79)	68 (76)	40 (58)	201 (73)	172 (63)	**0.006**	
3–12 (psychological distress)		98 (24)	15 (16)	32 (21)	22 (24)	29 (42)	73 (27)	99 (37)		
How often are you stressed?	419								**< 0.001**	
Never		14 (3)	6 (6)	4 (3)	3 (3)	1 (1)	9 (3)	5 (2)		
Sometime/month		156 (37)	49 (51)	53 (34)	35 (38)	19 (26)	122 (43)	56 (20)		
Some days/week		113 (27)	15 (15)	51 (32)	25 (27)	22 (30)	80 (28)	108 (38)		
Some days/week		89 (21)	18 (19)	32 (20)	18 (20)	21 (29)	47 (16)	69 (24)		
Most of the days/week		47 (11)	9 (9)	18 (11)	10 (11)	10 (14)	28 (10)	47 (16)		
Education in sport psychology, yes	418	180 (43)	29 (30)	67 (42)	44 (48)	40 (55)	145 (44)	133 (47)	0.827	
Yes, in school		46 (11)	8 (8)	19 (12)	14 (15)	5 (7)	31 (11)	26 (9)		
Yes, with the team		85 (20)	13 (13)	29 (18)	20 (22)	23 (32)	60 (21)	72 (25)		
Yes, private		18 (4)	5 (5)	9 (6)	3 (3)	1 (1)	9 (3)	8 (3)		
Yes, other		9 (2)	1 (1)	3 (2)	3 (3)	2 (3)	8 (3)	9 (3)		
Several options		22 (5)	2 (2)	7 (4)	4 (4)	9 (12)	18 (6)	18 (6)		
Regular contact with sport psychology consultant, yes	415	30 (7)	7 (7)	17 (11)	3 (3)	3 (4)	19 (7)	16 (6)	0.711	
Yes, in school		6 (1)	1 (1)	5 (3)	0 (0)	0 (0)	6 (2)	2 (1)		
Yes, with the team		9 (2)	1 (1)	6 (4)	1 (1)	1 (1)	5 (2)	5 (2)		
Yes, private		10 (2)	3 (3)	6 (4)	1 (1)	0 (0)	5 (2)	6 (2)		
Yes, other		2 (1)	2 (2)	0 (0)	0 (0)	0 (0)	1 (0)	0 (0)		
Several options		3 (1)	0 (0)	0 (0)	1 (1)	2 (3)	2 (1)	3 (1)		
*Brief COPE: adaptive behaviours (0–3)*										
Active coping	419	3.0 ± 0.7	2.9 ± 0.8	2.9 ± 0.7	3.1 ± 0.7	3.0 ± 0.7	3.0 ± 0.7	3.0 ± 0.8	0.463	−0.04
Emotional support	419	2.9 ± 0.9	3.0 ± 0.9	2.8 ± 0.9	3.0 ± 0.8	2.9 ± 0.8	2.9 ± 0.9	2.9 ± 0.8	0.223	−0.07
Instrumental support	419	2.8 ± 0.9	2.7 ± 0.9	2.7 ± 0.9	2.9 ± 0.8	2.8 ± 0.8	2.8 ± 0.8	2.8 ± 0.8	0.295	−0.06
Positive reframing	419	2.4 ± 0.8	2.4 ± 0.8	2.3 ± 0.8	2.5 ± 0.9	2.3 ± 0.8	2.4 ± 0.8	2.4 ± 0.8	0.801	−0.02
Planning	414	2.6 ± 0.8	2.6 ± 0.8	2.5 ± 0.8	2.8 ± 0.7	2.7 ± 0.7	2.7 ± 0.8	2.7 ± 0.7	0.361	−0.06
Humour	389	1.9 ± 0.9	1.7 ± 0.8	1.8 ± 0.9	2.0 ± 0.9	2.3 ± 1.0	1.9 ± 0.9	2.2 ± 1.0	**< 0.001**	0.38
Acceptance	417	2.7 ± 0.7	2.6 ± 0.8	2.7 ± 0.8	2.7 ± 0.7	2.7 ± 0.6	2.7 ± 0.8	2.6 ± 0.7	0.063	−0.11
Religion	389	1.1 ± 0.4	1.2 ± 0.5	1.2 ± 0.5	1.1 ± 0.3	1.1 ± 0.3	1.1 ± 0.4	1.2 ± 0.5	0.464	0.05
*Brief COPE: maladaptive behaviours (0–3)*										
Self- distraction	418	2.5 ± 0.6	2.4 ± 0.7	2.5 ± 0.6	2.6 ± 0.6	2.7 ± 0.5	2.5 ± 0.6	2.6 ± 0.7	0.101	0.10
Denial	418	1.4 ± 0.6	1.4 ± 0.5	1.5 ± 0.7	1.5 ± 0.7	1.4 ± 0.6	1.5 ± 0.7	1.5 ± 0.6	1.000	0.00
Substance	417	1.1 ± 0.3	1.1 ± 0.3	1.1 ± 0.3	1.1 ± 0.3	1.0 ± 0.0	1.0 ± 0.2	1.0 ± 0.2	0.549	–0.04
Behavioural disengagement	418	1.5 ± 0.7	1.5 ± 0.5	1.6 ± 0.7	1.5 ± 0.8	1.5 ± 0.6	1.5 ± 0.6	1.6 ± 0.7	**0.006**	0.17
Venting	419	2.4 ± 0.9	2.4 ± 0.9	2.3 ± 0.9	2.4 ± 0.9	2.4 ± 0.8	2.4 ± 0.9	2.4 ± 0.8	0.518	0.04
Self-blame	419	2.4 ± 0.9	2.2 ± 0.9	2.3 ± 0.9	2.5 ± 0.8	2.8 ± 0.7	2.4 ± 0.9	2.6 ± 0.8	**< 0.001**	0.34
*Passion Scale (0–7)*										
Harmonious passion	415	5.7 ± 0.9	5.7 ± 0.9	5.8 ± 0.8	5.7 ± 1.0	5.6 ± 0.9	5.8 ± 0.9	5.6 ± 1.	**0.002**	−0.19
Obsessive passion	417	4.8 ± 1.3	4.7 ± 1.4	4.8 ± 1.3	4.7 ± 1.3	5.1 ± 1.2	4.9 ± 1.3	4.8 ± 1.5	0.691	−0.02

Values are reported as means ± standard deviations or n (%). *P* values in bold type are significant. Effect size measured as Cohen’s *d*, where *d* = 0.2 indicates a small effect, *d* = 0.5 indicates a medium effect, and *d* = 0.8 indicates a large effect

^a^Missing value from 0–7 players for the different questions both at baseline and at follow-up. In the General Health Questionnaire, 12–15 missing answers at baseline; in Brief COPE humour and religion, 20 missing answers at baseline and 3 missing answers at follow-up

## Discussion

The main findings were that many adolescent female football players skip breakfast and lunch, have insufficient sleeping habits, experience stress and are classified as having psychological distress.

It was notable and worrying that more than 1 in 10 youth athletes skipped breakfast and lunch several times per week or every day. Breakfast and lunch are important meals for a growing adolescent football player. The questions about eating habits were not detailed, but a previous study on adolescent elite athletes (48% females, age 17 ± 0.9 years) showed that a high proportion did not meet national dietary recommendations regarding fish and vegetable intake [4]. Reaching the recommended nutritional intake reduced the odds of injury by 64% in adolescent athletes and therefore there is an urgent need to educate all sports entities i.e., coaches and their staff and athletes regarding adequate nutrition intake [4].

Few players used tobacco and alcohol in our young cohort, but drinking alcohol seemed to increase with age. In general, adolescents competing in sport at high levels are less likely to use tobacco and alcohol compared with adolescents not participating in competitive sports [18]. A plausible reason could be that athletes have more positive role models than nonathletes and a greater awareness of the detrimental effect on health and performance using tobacco and alcohol [18].

Overall, nearly 25% of the players took any medicine, which increased to 34% at the 1-year follow-up; 11% reported taking medication for asthma/allergy and 7% reported intake of pain killers. The use of medicine increased with age, mostly because of birth control pills and pain killers. In a previous study, the prevalence of allergic diseases was 35% in young male football players in the ages of 8–20 years, and 32% in controls; β_2_ agonists and inhaled corticosteroids were used in 7% and 11% of the football players, whereas only 4% of the controls were using these drugs [19]. The authors of that study speculated whether this discrepancy in medicine intake was due to better medical and disease management in athletes or a more general tendency to counteract symptoms to achieve better performances [19]. Medical screening to improve management is suggested in young football players, because respiratory allergies and exercise-induced bronchoconstriction seem to be underdiagnosed and undertreated in young football players [20].

Many players reported that they slept less than 8 h/night, had impaired sleep with daytime consequences, and stated that they were tired during the day every day or several days per week. These findings are in line with a previous report on elite adolescent athletes (48% females, age 17 ± 0.9 years) in which where 19% did not get the recommended amount of sleep during weekdays [4]. Appropriate sleep duration is between 9 and 11 h per night at 6–13 years of age and between 8 and 10 h per night at 14–17 years of age [21]. However, one study found that adolescent male football players reported better sleep measured with both quantitative and qualitative dimensions of sleep compared with controls [22]. Thus, we do not know if our adolescent female football players report better or worse sleep habits than a general population or compared with adolescent male players. Sleep is important for general health, daily functioning, performance, and well-being, and to habitually sleep outside the normal range may be a risk for serious future health problems such as heart disease, diabetes anxiety and obesity [21]. Adolescent athletes sleeping more than 8 h per night had lower odds of injury by 61% [4]. Therefore, it is important to observe adolescents with impaired sleep and identify possible strategies for intervention to prevent future health problems.

A large proportion of players had high scores for psychological distress and stated that they were stressed some or most days/week, and the numbers even increased at follow-up. However, in a previous study on students aged 11–15 years, the mean GHQ score was 11.4 [15], which is comparable with our results. Negative stressors (e.g., health problems, parental conflicts, peers or friends, romantic problems) and positive stressors (e.g., leisure, school, receiving help, romance, and friendship) are common and intense among adolescents [23]. In a previous study, adolescent female athletes reported significantly higher self-perceived stress than male athletes [4]. Stressors play a crucial role in ways that may be deleterious for young people’s psychological and behavioural adaptation to society [23]. High stress levels have also been reported to be a predictor of sustaining injuries among athletes [5, 24], with less recovery and subsequent sport performance [6]. Therefore, it is important to be aware of stress levels in adolescent female football players to support and teach stress management.

Coping strategies, where participants are requested to indicate how they behave in different stressful situations, were reported. Generally, our players scored higher in all categories compared with youth and adult Swedish track and field athletes [25]. The score for adaptive behaviour (humour), maladaptive behaviour (behavioural disengagement and self-blame), reflecting negative thinking [25], increased slightly at the 1-year follow-up, but with small to medium effects. Using self-blame as a coping behaviour was related to overuse injuries in track and field athletes [25]. Maladaptive coping strategies have been evaluated as risk factors for injury in football players, but showed no association [5, 24].

Passion for sport is divided into harmonious and obsessive passion. Harmonious passion is positively associated with both flow and psychological well-being, and obsessive passion is negatively associated with psychological well-being [26]. Our players scored higher in harmonious passion with a minimal decrease in the scores at the 1-year follow-up. They had relatively high scores on obsessive passion (mean, 4.8). An association between greater obsessive passion (3.2 vs. 2.7) and injuries in runners has been reported [27] and may predispose athlete to overuse injuries [28]. Therefore, it could be important to analyse the players’ passion for football.

Systematic injury prevention training is increasingly in focus. In our cohort, 76% stated that they regularly performed some kind of injury prevention training (e.g., Knee Control, HarmoKnee or FIFA 11+) [11]. Such neuromuscular training programmes have been shown repeatedly to reduce the risk of injury in football players, with greater reductions in injuries when a larger number of training components are included [11]. Therefore, it is notable that the proportion of players who stated that they took part in prevention training decreased from 78 to 70% at the 1-year follow-up. However, the decrease was only 24 players in absolute numbers and the frequency of using the programmes increased slightly. About half of the players had trained and played matches with older players in the previous 6 months. Playing with older players has been suggested as a risk factor for sustaining a knee injury in particular, but there is conflicting evidence and more research is needed before any conclusions can be drawn [29, 30].

Nearly half of the players stated that they had received education in sport psychology, but as expected, few players in this cohort had regular contact with a sport psychology consultant. Most of the players had contact through the team or school and thus probably not with a qualified sport psychologist. The psychological aspects in training, including self-efficacy, self-confidence, and motivation, are highlighted more and more, not just physical demands such as agility, muscular strength and aerobic capacity. Cognitive strategies, and particularly imagery, appear to improve football performance, and younger players use such techniques to a greater extent than older football players [31].

Generally, our results show that many adolescent female football players skip meals, have insufficient sleeping habits, experience stress and are classified as having psychological distress, which coaches and clinicians should be aware of. Therefore, it is important to catch signs of stress and illness among the players through conversations and simple estimation instruments, and possibly adapt training and match load. It is also important to have the diagnosis of relative energy deficiency in sport (RED-s) in mind. The diagnosis requires a low threshold of suspicion and questions about e.g. diet, weight and sleep changes, training hours, stress, mood, and amenorrhea should be asked [32]. Presence of amenorrhea for > 3months is the first manifestation of menstruation dysregulation [33]. In our cohort, 97% of the females in the ages of 15–17 years stated that have had their menarche, but 22% reported amenorrhea for more than 3 months in a row after their menstrual bleedings had become regular. Educational meetings for athletes, parents and to all sports entities i.e., coaches and their staff about stress, sleep, diet, etc. could be arranged. For athletes who may need more professional support, other additional interventions could be recommended. A multidisciplinary treatment approach, including medical, dietary, and mental health support, is necessary to treat RED-s or functional hypothalamic amenorrhea [32, 33]. In general, we found small differences in estimates between baseline and follow-up with small effect sizes especially in Brief COPE and the Passion Scale. Other changes between baseline and follow-up are probably due to the age e.g., body mass, training with older players, other training (not football), and drink at least one glass of alcohol.

The main strengths of this study are the large sample from the Stockholm football district, few missing data, and a relatively high follow-up rate. However, our aim was to recruit players from the 2 highest divisions in the age groups and from the main cities in Sweden. Because of limited resources, the sample was mainly from the Stockholm region. We chose to include clubs in the 2 highest divisions in each age group to get a high level of follow-up with less players quitting football. Therefore, we do not know if these results are generalizable to all playing levels. Other limitations are the extensive questionnaire with the risk of exhaustion, and there is also a risk for misclassification in a cohort of adolescent players. Most of the players filled in the questionnaire at home, although some completed it at the football academy. Therefore, we are not aware of the influence of parents and team mates on the responses. Another limitation is that not all questions and questionnaires are validated for adolescents; for example, the Stockholm Public Health Cohort survey [7] and the sleep questions [13].

Characteristics of lifestyle factors in adolescent female football players could be used as normative values, and their potential influence on performance, well-being and risk of injury should be explored further. In conclusion, many adolescent female football players skip breakfast and lunch several days/week or every day, have insufficient sleep, experience stress and tiredness with daytime consequences and are classified as having psychological distress. These factors increased over 1 year.

## Data Availability

De-identified data are available from the first author (A.F.) upon reasonable request.

## Abbreviation

GHQ: General Health Questionnaire
